# Dendritic Cell-Based Vaccine Efficacy: Aiming for Hot Spots

**DOI:** 10.3389/fimmu.2015.00091

**Published:** 2015-03-03

**Authors:** Gabriela Andrea Pizzurro, María Marcela Barrio

**Affiliations:** ^1^Centro de Investigaciones Oncológicas – Fundación Cáncer (CIO – FUCA), Buenos Aires, Argentina

**Keywords:** immunotherapy, cancer vaccines, dendritic cells, vaccine injection site, draining lymph nodes, antitumor T cells

## Abstract

Many approaches for cancer immunotherapy have targeted dendritic cells (DCs), directly or indirectly, for the induction of antitumor immune responses. DC-based vaccines have been developed using a wide variety of *ex vivo* DC culture conditions, antigen (Ag) source and loading strategies, maturation agents, and routes of vaccination. Adjuvants are used to activate innate immune cells at the vaccine injection site, to promote Ag transport to the draining lymph nodes (LNs) and to model adaptive immune responses. Despite years of effort, the effective induction of strong and durable antitumor T-cell responses in vaccinated patients remains a challenge. The study of vaccine interactions with other immune cells in the LNs and, more recently, in the injection site has opened new doors for understanding antitumor effector T-cell licensing and function. In this review, we will briefly discuss the relevant sites and up-to-date facts regarding possible targets for antitumor vaccine refinement. We will focus on the processes taking place at the injection site, adjuvant combinations and their role in DC-based vaccines, LN homing, and modeling vaccine-induced immune responses capable of controlling tumor growth and generating immune memory.

## Introduction

Therapeutic cancer vaccines are the focus of active investigation and testing, fueled by their promise as a tool for cancer treatment. In contrast to other cytotoxic therapies, cancer vaccines have demonstrated minimal toxicity in clinical trials to date and have showed some positive results, including slowed tumor growth rates and improved overall survival, but no substantial reductions in tumor burden or improvement in relapse-free survival (1, 2). Dendritic cells (DCs) are professional antigen (Ag)-presenting cells (APCs) that, upon activation, can initiate and direct Ag-specific immune responses. DCs have become a promising tool for cancer immunotherapy due to considerable advances related to their biology and their role in T-cell activation, which has clearly opened avenues for the development of vastly improved clinical protocols (3). Roads are currently leading to: (a) the optimization of DC vaccines to elicit strong and long-lived Ag-specific cluster differentiation (CD)8^+^ and CD4^+^ T-cell immunity to control early stage disease and (b) the development of strategies that combine highly immunogenic DC-based vaccines and immunomodulatory antibodies for advanced disease, both of which enhance the potency of beneficial immune arms and offset immunoregulatory pathways (4, 5). Cancer vaccination efforts are centered on the disruption of the tolerogenic state of the immune system and direction of an effector T-cell (Teff) response, ultimately leading to cancer regression. This latter point remains a significant challenge when it comes to an objective beneficial outcome in patients. Several questions remain open on the true relevancy of DC-based vaccination in the clinic and the lessons learned over years of clinical investigation (3, 6, 7). Despite their complexity, models reflecting experimental results have tried to simplify the system to test immunotherapy treatment protocols with *in silico* simulations of vaccine efficacy (8). However, in addition to multiple parameters in vaccine design, intrinsic variables present in individual patients may equally influence the elicited immune response. Here, we will briefly discuss the main critical stages where immune response can be modulated, and the different factors affecting DC-based strategies, which have been obtained in recent years from basic studies of murine and human DC–T-cell interactions, animal models, and human preclinical and clinical studies.

## DC-Based Vaccines: Current Approaches

The application of *ex vivo*-educated DCs emerged in an effort to avoid possible interferences in therapeutic efficacy due to the dysfunction of endogenous DCs commonly observed in cancer patients (9–11). *Ex vivo* DCs are mainly generated through *in vitro* differentiation of peripheral blood mononuclear cells (PBMCs) in the presence of granulocyte–macrophage colony-stimulating factor (GM-CSF) and interleukin (IL)-4 or IL-13 (12). DC-based vaccines should present a “mature” state in order to activate an Ag-specific immune response upon T-cell encounter. This differentiated state is characterized by the expression of several costimulatory molecules, the necessary activating second signal in the immunological synapse (13). They include CD80 and CD86, CD40, CD70, or inducible T-cell costimulator ligand (ICOS-L) molecules, which interact with their counterparts CD28, CD40L, CD27, and ICOS, respectively, expressed by T cells. In addition, DCs have elevated levels of Ag-presenting molecules, i.e., major histocompatibility complex (MHC) class I, MHC class II, and CD1 molecules. An immunostimulatory cytokine profile is also required to trigger an efficient CD8^+^ T-cell response, currently considered as the “third signal” (9, 14). This process is accompanied by an augmented chemokine-driven migratory capacity, with increased chemokine receptor 7 (CCR7) expression, which favors lymph node (LN) homing and T-cell encounter and allows Ag presentation and T-cell activation (15). This complex context has required the exploration of various strategies (16). A “standard” maturation cocktail, comprised of tumor necrosis factor (TNF)-α, IL-1β, IL-6, and prostaglandin E2 (17) has been extensively used to develop conventional DCs. This “standard” mature DCs acquire an activated phenotype, respond to LN homing signals, and secrete moderate amounts of T helper (Th)1 cytokine IL-12p70, but with low immunoregulatory cytokine production (17). Targeting the innate danger signal pathway of toll-like receptors (TLRs) improved migration, cytokine profiles, and immune responses (18–20). Alternative tracks use type-1 polarized DCs, generated in the presence of interferon (IFN)-γ, which show a mature state with IL-12p70 release, chemotactical response to the LN homing chemokine CCL19, and generate Ag-specific Teff (21, 22). “*Fast* DCs,” which are generated in a 3-day culture, show similar performances (23, 24). Taken together, considerable progress has been made over the years, although the potential impact of *ex vivo*-generated DCs on immunotherapy requires additional studies to be fully disclosed.

The production of *ex vivo*-generated DCs for personalized vaccines is associated with several inconveniences. The time-consuming vaccine preparation and elevated costs of production have led to the study of alternative, but related strategies. *In vivo* DC activation and Ag loading are an interesting approach, as it by-passes the *ex vivo* DC vaccine drawbacks and may combine the benefits of the physiological environment, making selective use of all the DC subsets present in the dermis and epidermis (25–28). Some targeted and non-targeted vaccines are poorly immunogenic when applied alone. The addition of adjuvants has generated a more favorable environment with viable and motile cells available to initiate a successful immune response, rather than an inflamed Ag depot (29). Many adjuvants currently under evaluation as constituents of cancer vaccines proved to be more than mere delivery systems. Mineral salts, emulsions, and liposomes were able to trigger B-cell and Th1- or Th2-polarizing immune responses. Immunostimulant adjuvants, like TLR-ligands, cytokines, saponins, and bacterial exotoxins, have components that directly interact with the immune system to intensify the elicited response. These events are reviewed in detail by Dubensky and Reed (30). Due to high side effect and toxicity risks with relative low benefit, increased regulatory standards have imposed several barriers for the approval of new adjuvants that must be overcome to meet the increasing demand.

## Vaccine Administration: Immune Role for the Injection Site

The route of DC-based vaccine administration remains a matter of debate. Maintaining DC viability and maturation status while eliciting a T-cell response can be difficult due to technical and/or budget limitations (31, 32). Direct Ag delivery to DC through selective targeting using monoclonal antibodies against endocytic receptors, such as the C-type lectin receptor DEC205, results in 100-fold more efficient CD4^+^ and CD8^+^ T-cell activation than fluid-phase or solute pinocytosis (33, 34). However, DC-targeted vaccines must be timely combined with adjuvants in order to avoid Ag-specific tolerance (35). Targeting the skin either with *ex vivo*-loaded DCs or *in vivo* DC-based strategies has improved immune responses. Local delivery of cell-associated Ag showed delayed T-cell cross-priming, but a more robust, polyfunctional, effector response (36). Likewise, intradermal (i.d.) administration induces a more potent and long-lasting specific, functional CD8^+^ T-cell response that effectively breaks self-tolerance in mice (37) and provides a superior functional tumor Ag-specific reaction in delayed-type hypersensitivity sites after DC vaccination in melanoma patients (32). The skin offers a rich immune network comprised of Langerhans cells in the epidermal compartment and dermal DCs. Local APC are accompanied by specialized cells with immune function, including macrophages, keratinocytes, mast cells, natural killer (NK) T cells, and fibroblasts, with access to draining lymphatic and blood vessels (27). These features turn the skin into an ideal niche for DC-based vaccination, so far extensively explored in mice (25, 38) and in *in vitro* human cell cultures (39), although little is known about human APC function *in situ*. Microneedle arrays are a new vaccine delivery system that can enter the skin at a very low insertion force and controlled depth, facilitating i.d. vaccine administration in simultaneous proximal inoculations (40). The microneedles offer a dose-sparing advantage, improved safety, and patient compliance, and therefore stand firm as an effective, easy delivery route with a great future in vaccination (41). Polymeric, water-soluble microneedles dissolve and release nanoencapsulated Ag into skin tissue, with no residual waste. In mice, skin-derived DCs delivered nanoparticles to draining LNs, subsequently inducing a potent activation of specific CD4^+^ and CD8^+^ T cells (42); however, the study did not make comparisons to traditional Ag delivery systems.

The natural skin micromilieu should not be underestimated when choosing the vaccine injection site, as it may influence the elicited immune response, especially with respect to tumor localization (43, 44). Keratinocytes may also be involved in local APC inflammatory activation through the secretion of the active form of IL-1β, as well as in suppressive imprinting through transforming growth factor (TGF)-β1 production. Therefore, the immunological balance within the newly formed structure at the site of DC-based vaccine injection, observed both in mice (45) and humans (46), must be delicately studied. One study reports that repeated vaccination with melanoma peptides in incomplete Freund’s adjuvant (IFA) induced organized and persistent lymphoid aggregates in the patients’ dermis. They contained separate B- and T-cell areas, with proliferating CD4^+^ and CD8^+^ T cells, as well as CD4^+^FoxP3^+^ lymphocytes, mature DC, high endothelial venule-like vessels, and lymphoid chemokines (46). Though Ag persistence at the vaccination site produces a delayed, but stronger Teff response (36), the long-persistent peptide depots with IFA induce tumor-specific CD8^+^ T cells that remain locally sequestered, dysfunctional, and eventually deleted, rather than redistributing into the tumor, resulting in hyporesponsiveness to subsequent vaccination (29). Biomaterials may overcome these limitations, as short-lived formulations like hydrogels can provide a niche that allows *in situ* priming and immune modulation while preserving cell viability, thus enhancing the efficacy of next-generation immunotherapy (47, 48).

The recruitment of APC to the injection site and subsequent local activation is a thoroughly explored strategy. Different chemoattractants such as GM-CSF and chemokines have been used as adjuvants in the clinical setting (49), with occasionally unexpected results. Conditioning the injection site with macrophage inflammatory protein (MIP)-3α-expressing irradiated cells prior to DC vaccination effectively suppresses B16F1 melanoma growth in animals (50). However, co-expression of MIP-1α nullified the GM-CSF-induced immune response against the GL261 glioma, rather than attracting T cells to GM-CSF-stimulated DC (51). TLR-ligands are added to vaccine formulations to avoid Ag-specific tolerance, i.e., when targeting DEC205 (35), and to further stimulate cells of the innate immune system, thereby increasing the potency of the elicited immune response (52). Their ability to skew the response toward a Th1 or Th2 profile, generating different Ag-specific Teff to regulatory T cell (Treg) ratios, is the main parameter evaluated, and differs according to vaccine setting. Some TLR2/4, TLR3, and TLR9 ligands, like Bacillus Calmette–Guerin, polyinosinic:polycytidylic acid, and CpG oligodeoxynucleotides respectively, are currently being studied in DC-based cancer immunotherapy with combinatorial positive results (19, 53–55), while the TLR7 ligand imiquimod and lipid mediators such as QuilA or ISCOM produce more varied results (53, 54, 56–58).

## Draining LN: DC Migration and the Activation of T Cells

After vaccine administration, activated DC must closely interact with *naïve* T cells, which, upon Ag recognition, exert their cytotoxic, helper, or regulatory function. The LN is a multifunctional and compartmentalized organ that collectively offers structural guidance for optimal Ag-loaded DC proximity to, and scanning of a large number of T cells (59, 60). Apart from the events controlling DC migration from the skin toward, into, and within the lymphatic vessels (61), recent advances in multiphoton-based time-lapse and intravital microscopy have provided insight into the complex migratory behavior and interactions of DCs and T cells within the lymphoid microenvironment, mainly in mice. Activated DCs typically arrive at the draining LN between 24 and 72 h after injection, but it can be as soon as 2 h after stimulation (62). DCs vigorously extend long, agile dendrites, thus promoting the scanning of a vast, autonomously moving T-cell repertoire. CD4^+^ and CD8^+^ T cells have different Ag surveillance strategies, presenting asymmetric roles for MHC interactions and LN transit times (63, 64). Ag-bearing DCs are highly efficient recruiters of peptide-specific T cells, in part through the secretion of chemokines. The resulting overall avidity of the interaction influences the probability that T cells are stably captured by DCs (65, 66). Chemokines present in the LN structure promote DC interaction with cognate CD4^+^ and CD8^+^ *naïve* T cells (59, 67) that, once activated, leave the LN to exert their function. Taken together, these complex cellular behaviors promote proper Ag presentation in the LN and the potential efficient systemic antitumor CD8^+^ T-cell response.

The efficiency of DC migration to the LNs has been related to their maturation state (68) as well as to the expression of CCR7, which confers additional attributes to mature DC, such as migratory speed and inhibition of apoptosis (69, 70). Again, the route of administration has great influence over both DC migration and activated T-cell LN homing, and therefore may improve clinical outcome, as seen in mice (31). When choosing i.d. or subcutaneous (s.c.) inoculation, the manipulation of either the injection site or the DCs themselves can stimulate DC migration. As mentioned before, conditioning of the injection site can activate resident APC and generate a more immunogenic environment and more permeable lymphatic vessels with increased secretion of the CCR7-ligand CCL21 (71, 72). DC mobilization can be improved through metalloproteinase (MMP) secretion and changing the adhesion molecule profile (73, 74). In mice, increasing the number of injected DCs has shown improved migration efficacy. CCR7^+^ DCs efficiently induce a rapid increase in LN cellularity, observed before the onset of T-cell proliferation. The elicited CD4^+^ T-cell response is proportional to the number of Ag-carrying DCs reaching the LN (71). In patients, decreasing the number of injected DCs actually improves the proportion of cells migrating to the LNs, but the analysis is limited by the detection method (75). Today, the number of Ag-loaded DCs required at the LN to mount a complete antitumor response is unknown.

Maximizing LN homing of DC-based vaccines may enhance antitumor responses (76), therefore techniques for the clinical assessment of DC migration have been perfected, including fluorescent-, ^111^Indium-, and magnetic particle-labeling for cell tracking (77, 78). But, more importantly, DCs must not only reach one or multiple secondary lymphoid tissues, but also enter the T-cell-rich areas in order to efficiently elicit an antitumor CD8^+^ response that may contribute to a better clinical outcome. Furthermore, following the vaccination of tumor-bearing mice, high tumor-specific Teff to specific Treg ratios in draining LN were associated with enhanced CD8^+^ T-cell infiltration and durable rejection of tumors (53). The normal immunological function of regional tumor-draining LN is compromised through immunosuppressive mechanisms (79) and the presence of metastases, though they remain a rich source of sensitized T cells (43, 80, 81). FoxP3^+^ T cells induce the death of DCs and impede normal motility and the cross-priming of CD8^+^ T cells (82). Spatial organization of DCs within the tumor-draining LNs impacts the duration of disease-free survival in breast cancer patients, where the number and size of DC clusters were associated with DC maturation status and T-cell co-localization and interaction (83). Therefore, knowing which DC subsets are present in LNs and their role in T-cell activation (84), it is possible to specifically target therapies to break the tolerogenic environment. Combinatorial approaches may include the use of adjuvants or cytokines (81, 85) as well as other cells expressing costimulatory molecules or functioning as helpers, such as NK cells (58, 86–88).

## Effector Antitumor Immune Responses: Breaking the Ice

The efficacy of antitumor therapy mainly depends on four critical components: the elicited CD8^+^ Teffs, the quality of the CD4^+^ helper T cells, the elimination and/or non-activation of Tregs, and the breakdown of the immunosuppressive tumor microenvironment (3). Therapeutic vaccination is currently designed as an adjuvant or neoadjuvant treatment for patients with a high risk of recurrence. Adequate vaccine design and a better understanding of host–tumor interactions are needed to overcome systemic and local immune tolerance and generate an effective antitumor response (2). There are several essential steps in vaccine formulation that collectively impact the immune response and ultimately the clinical outcome. Vaccine design must consider the administration route (36), the type and amount of Ag provided (89–92), the delivery system (93), and the addition of different immunostimulants that lead to *in vivo* activation of CD8^+^ T cells (53, 58) as well as long-term memory (54). Favorable clinical responses require a Th1 immune profile, and furthermore, a high vaccine Ag-specific Teff to Treg ratio was predictive of clinical benefit (94). Along with CD8^+^ Teffs, CD4^+^ T cells strongly influence the elicited antitumor response. Ag-loaded DCs can induce human CD4^+^ T-cell proliferation that combined with strong activating signals, overcome immunosuppression through Th17 differentiation (95). In mice, CD4^+^ T cells generate increased numbers of tumor-specific effector and memory CD8^+^ T cells. The role of CD4^+^ T cells is critical in the early stages of the immune response, helping reduce CD8^+^ T-cell exhaustion by decreasing expression of the immunoinhibitory receptor programed death (PD)-1 (96). However, selective CD4^+^ T-cell tolerance underlies ineffective vaccination. Vaccine-mediated *naïve* T-cell priming is inhibited due to a minor but distinct population of tumor-reactive CD4^+^ T cells, generated in the tumor-draining LNs and systemically redistributed (97). Higher numbers of administered DCs revert this effect, allowing CD4^+^ T-cell priming comparable to tumor-free mice (97). Incorporating CD4^+^ T-cell epitopes from foreign Ags into vaccines reconstitute CD4^+^ T-cell help, reactivating the latent functional capacity of Ag-specific CD8^+^ T- and B-cell pools with durable antitumor immunity (98). In melanoma patients, coactivating Ag-specific CD4^+^ T cells with MHC-I/II peptide-loaded DCs augments Ag-specific CD8^+^ T-cell responses, which contributes to improved clinical responses, as compared to dacarbazine-treated control patients (99).

Measuring the frequency of IFN-γ-secreting CD8^+^ T cells is insufficient to evaluate the quality of vaccine-elicited immunity (3). The localization of CD8^+^ Teff impacts their phenotype and function. Melanoma-infiltrating CD8^+^ Teffs express higher levels of PD-1 and cytotoxic T-lymphocyte-associated protein (CTLA)-4, both associated with T-cell exhaustion, than their counterparts in normal tissues and circulating blood (100). The tumor microenvironment exhibits several immunosuppressive factors, reviewed in Vasaturo et al. (101) that neutralize tumor-specific T cells and hamper DC vaccination efficacy. Along with myeloid-derived suppressor cells (MDSC), Tregs directly suppress CD8^+^ Teff responses at the tumor site. Increased numbers of MDSCs and Tregs are also found in the metastatic LN and peripheral blood of patients (102). Studies in mice have demonstrated a superior suppressive capacity in Ag-specific Tregs than polyclonal Tregs. Melanoma patients display a broad repertoire of circulating tumor Ag-specific Tregs that are not detected in healthy individuals (103). The use of HClO-oxidized tumor lysate for DC loading reduced circulating Tregs and serum IL-10 while eliciting a potent Teff response against ovarian tumor Ags, as compared to standard Ag preparation methods (104). Adjuvants can determine the Th response profile as well as the generation of MDSC and Tregs (53, 56). Vaccine antitumor effects can be improved substantially with combination therapy since it allows a simultaneous counterattack on multiple tumor evasion mechanisms. Radiation therapy modifies both the phenotype and the microenvironment of tumor cells, but requires CD8^+^ T cells to achieve a therapeutic effect. Further combination with Th1 cell therapy augmented the generation of infiltrating Teff and induced a complete regression of tumors in mice (105). Treg depletion helped induce protective antitumor immunity and the generation of immunological memory after s.c. peptide immunization (55). Blockade of PD-1 enhanced breast cancer vaccine efficacy by altering both the CD8^+^ T-cell and DC components of the tumor microenvironment (106). Peptides combined with a single low dose of cyclophosphamide reduced the number of Tregs in renal cell cancer patients (107). Nevertheless, combinatorial therapies must be carefully designed and tested due to possible increased toxicity, autoimmunity, or opposite effects, e.g., the systemic coadministration of IL-2 alongside DC vaccination resulted in higher Treg frequencies in peripheral blood and invariant Ag-specific Teff response (32).

## Concluding Remarks

**Figure 1:**
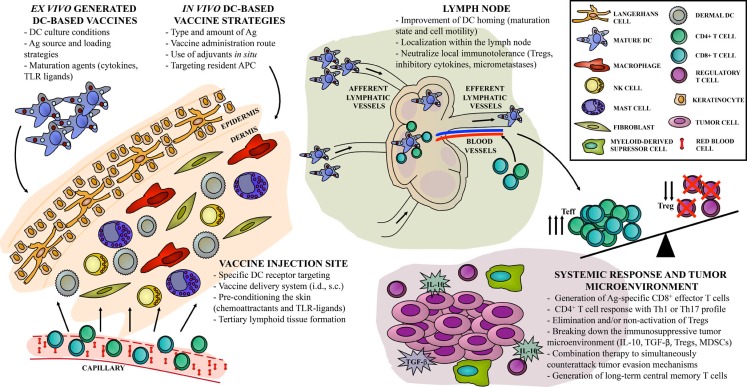
**“Hot spots” in DC-based vaccine design**. Current approaches for cancer immunotherapy targeting DC include multiple steps, resulting in one out of hundreds of possible combinations, with different antitumor immune responses. Direct or indirect strategies can be achieved either *ex vivo* or *in vivo*, with particular implications to be considered in each case. We identify the relevant sites, or “hot spots,” that become targets for antitumor vaccine refinement according to recent years of basic and clinical investigation. First, the skin as an injection site can be widely activated in order to improve vaccine efficacy. Second, DC-based vaccine migration to the lymph node compartment can be exploited to enhance the induction of Ag-specific immune effectors. Finally, systemic vaccine-induced immune response should overcome local immunosuppression to control tumor growth and to generate long-term immune memory.

There is no clear consensus for a “DC vaccine recipe” that would provide a better performance in terms of disease control. There are as many possible components for vaccine formulation as variations in vaccination scheme design. However, integrating an optimized vaccine preparation with a local immune activation seems to be the future of treatment platforms (Figure 1). There is a push for new immunomonitoring parameters, since assessment of relevant immune responses following DC-based vaccination remains a true pitfall. Events occurring in the tumor microenvironment are not accessible or are even invisible in undetected micrometastases. Traditional monitoring of peripheral blood immune response does not correlate with clinical outcome of therapeutic vaccines. Over the past decade, the increased knowledge of DC biology and crucial mechanisms involved in the generation of the immune response provided valuable tools to improve DC-based vaccines and position them as a potentially curative strategy for cancer patients. The discovery of molecular targets and blocking antibodies for immune checkpoints has opened new avenues for combination therapy with DC-based vaccines. In the near future, cancer immunology researchers face the challenge of integrating all of the knowledge and advances to design rational and efficient DC-based vaccine treatments to achieve long-term clinical response.

## Conflict of Interest Statement

The authors declare that the research was conducted in the absence of any commercial or financial relationships that could be construed as a potential conflict of interest.

## Abbreviations

Ag, antigen; APC, antigen-presenting cell; CCR7, chemokine receptor 7; CD, cluster differentiation; CTLA, cytotoxic T-lymphocyte-associated protein; DC, dendritic cells; GM-CSF, granulocyte–macrophage colony-stimulating factor; ICOS-L, inducible T-cell costimulator ligand; i.d., intradermal; IFA, incomplete Freund’s adjuvant; IFN, interferon; IL, interleukin; LN, lymph node; NK, natural killer; MDSC, myeloid-derived suppressor cells; MHC, major histocompatibility complex; MIP, macrophage inflammatory protein; MMP, metalloproteinase; PBMC, peripheral blood mononuclear cells; PD, programed death; s.c., subcutaneous; Teff, effector T cell; TGF, transforming growth factor; Th, T helper; TLR, toll-like receptor; TNF, tumor necrosis factor; Treg, regulatory T cell.
